# Content validation of educational materials on maternal depression in Nigeria

**DOI:** 10.1186/s12884-022-04575-5

**Published:** 2022-04-15

**Authors:** Adeyinka Olufolake Adefolarin, Asiki Gershim

**Affiliations:** 1 Department of Psychiatry, College of Medicine, University of Ibadan, Ibadan, Nigeria; 2 Department of Health Promotion and Education, faculty Public Health, University of Ibadan, Ibadan, Nigeria; 3 African Population and Health Research Center, Nairobi, Kenya; 4 Department of Women’s and Children’s Health, Karolinska Institute, Stockholm, Sweden

**Keywords:** Content validation, Educational materials, Maternal depression, maternal-child health, information, education and communication materials, Health promotion and education

## Abstract

**Background:**

This study describes the content validation process of the already developed English and Yoruba (poster and leaflet) and Yoruba (song) maternal depression educational materials in Nigeria.

**Methods:**

This study is a cross sectional study which is a part of a larger study on training and supervision of Primary health care workers. Study utilized health professionals’ judgement for content validation, and maternal-child health clients’ evaluation for face validation with the use of Suitability Assessment of Materials (SAM). Six bilingual professionals validated both English and Yoruba version of materials (Song has only Yoruba version) and 50 clients evaluated each Yoruba material. Validity Index was calculated by formula and inter-rater agreement using intra-class coefficient (ICC) was analyzed on Professionals’ ratings. ICC, ‵t′ test and Pearson correlation were analyzed on professionals’ rating versus randomly selected six clients’ rating. Descriptive statistics, and fisher exact test were used for other statistical analysis with SPSS version 25.

**Results:**

The mean age of the professionals for poster was 44.3 ± 6.0 years, for leaflet 39.8 ± 7.2 years, for song 43.8 ± 8.4 years. For maternal child health clients, mean age is: 30.7 ± 5.4 years for poster; 31.3 ± 5.2 for leaflet and 29.0 ± 5.1 for song. Outcomes of bilingual professionals’ validation are validity index: English {leaflet (0.94), poster (0.94)}, and Yoruba {leaflet (0.94) poster (0.94) and song (1.00)}. More than 80% clients rated the suitability of each material as superior. There is no significant relationship between clients’ sociodemographic characteristics and their ratings across content, literacy demand and cultural appropriateness domains of the three materials on fisher exact test. The inter-rater agreement among the professionals is excellent on leaflet and song ICC > 0.8, but it is weak on the poster ICC < 0.6. There is no inter-rater agreement on all the three Yoruba materials, but a negative linear correlation was found on the leaflet between the professionals’ ratings and the randomly selected clients’ ratings. ‵t′ test found no statistical difference in the ratings of the professionals and clients only on song material.

**Conclusion:**

This study shows the process of validation of the English and Yoruba versions of the educational materials. This process should be leveraged in the content validation of other maternal-child health education materials in Africa.

## Background

Validation is a widely known scientific process and a quantifiable way of authenticating that a particular material contains adequate or appropriate content to perform the task or objective it is meant to perform/deliver [1]. It is widely used in the development of measuring scales or research questionnaire [2]. Professionals in the field of interest are recommended to participate in content validation while end users are recommended to participate in face validation [2]. The agreement of rating among the professionals is the strength of the validation index. The agreement can be in form of quantifying consensus (content validity ratio) [3] or measuring proportional agreement (content validity index) [4] measuring inter-rater experts’ agreement (Cohen’s coefficient kappa (k) or Intra class correlation) [5] 0r the use of Delphi technique [6] The application of content validation has been found to be relevant in most studies on Information Educational and Communication (IEC) materials [7, 8], and it is commonly conducted in studies from the high-income countries [9].

In the low- and middle-income countries (LMICs), print educational materials are widely used to address prevailing health conditions [10–12], but studies showing their development and validity processes are scare. Although, the use of targeted educational materials for health education is emphasized in the primary health care guideline in Nigeria [13], but not all prevailing health condition is being addressed in the primary health care routine health education. Maternal depression is one of them [14]. Maternal depression is a common condition which requires health education [15–17] recommended intense enlightenment through health communication to address maternal depression in LMICs. In line with this recommendation, and the misconceptions about maternal depression which is common among the Yoruba community in Nigeria [18], there was a need to develop and validate materials which provide correct health education on maternal depression.

So far, standard guidelines for validation of educational materials are scarce, but guideline on validation of scales and research instrument are available [2]. Studies which make use of methodological research designs are available. They show the details of process of development of educational materials and the validation with focus on either suitability [19–21]; relevance [7]; content and readability or understanding of audio-visual materials [22] and adequacy [8] . These studies have used various instruments including Suitability Assessment of Materials (SAM) [23], Comprehensibility Assessment of Materials CAM [24], Adapted Questionnaire [25] and Patient Educational Materials Assessment Tool (PEMAT) [22]. Studies from Nigeria often end the development of educational materials process at pretest, the materials are rarely validated [26].

In our study, we carried out content validation as found in literature [2, 7], and we validated print and song materials on maternal depression. The primary goal is to show the content validation process, and to make validated health education materials on maternal depression available for health educators in Nigeria and elsewhere.

## Methods

### Study design and setting

This is a cross sectional study embedded in a parent study which implemented and evaluated training and supervision of health talk delivery on maternal depression. The parent study was carried out among primary health care workers in Ibadan, Nigeria. This study was set to validate educational materials used for the parent study.

This validation study took place in two of five local government areas (LGAS) in Ibadan metropolis. Primary health clinics (phcs) were used in the two LGAs. Primary health clinics do not offer laboratory testing, 24 h service, minor surgery, and admission, in addition to the out-patient services. The primary health clinics serve up to 5000 people, and it only offers outpatient services. They are referral services for mobile health posts.

### The materials for validation

**Fig. 1:**
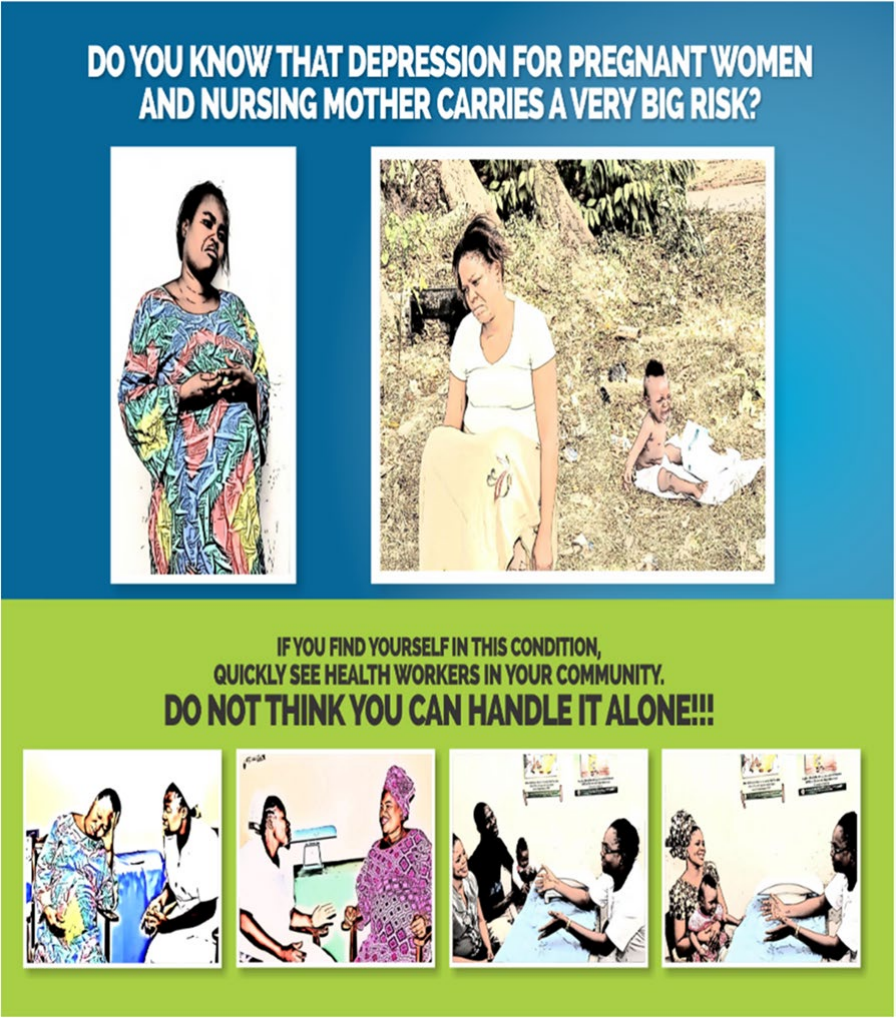
English poster on maternal depression

**Fig. 2:**
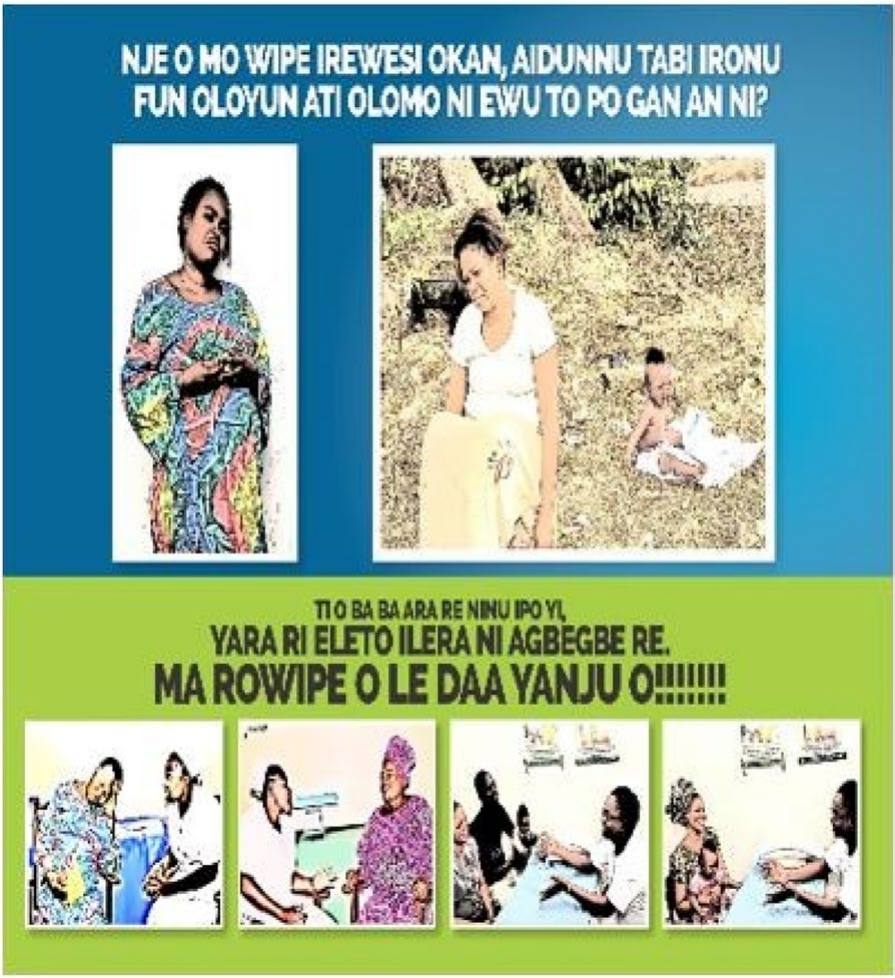
Yoruba poster on maternal depression

**Fig. 3:**
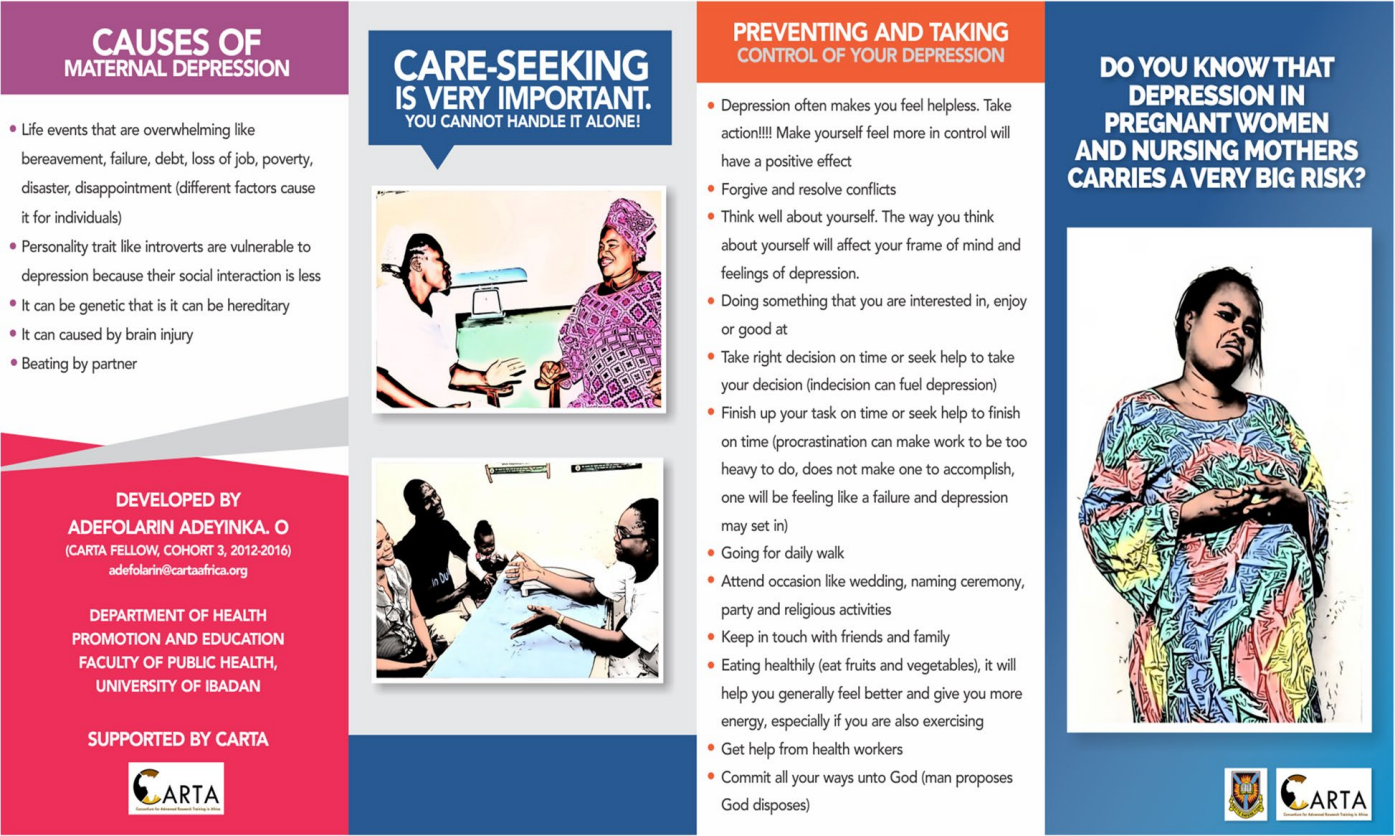
English leaflet on maternal depression on its front page. It shows the definition, symptoms, and consequences of maternal depression

**Fig. 4:**
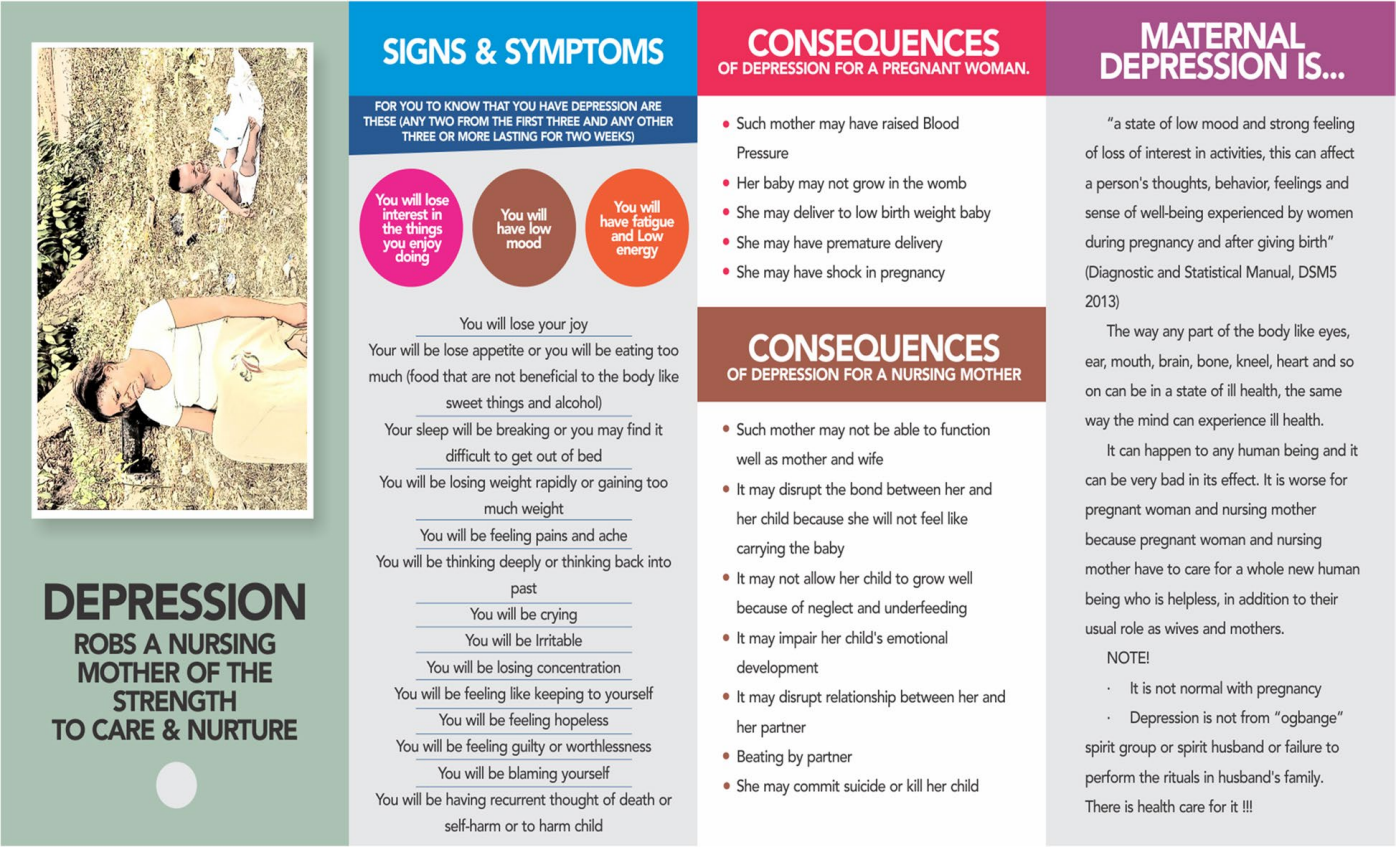
Yoruba leaflet on maternal depression. Is the back page of English leaflet on maternal depression. It shows how to prevent and to take control of depression

**Fig. 5:**
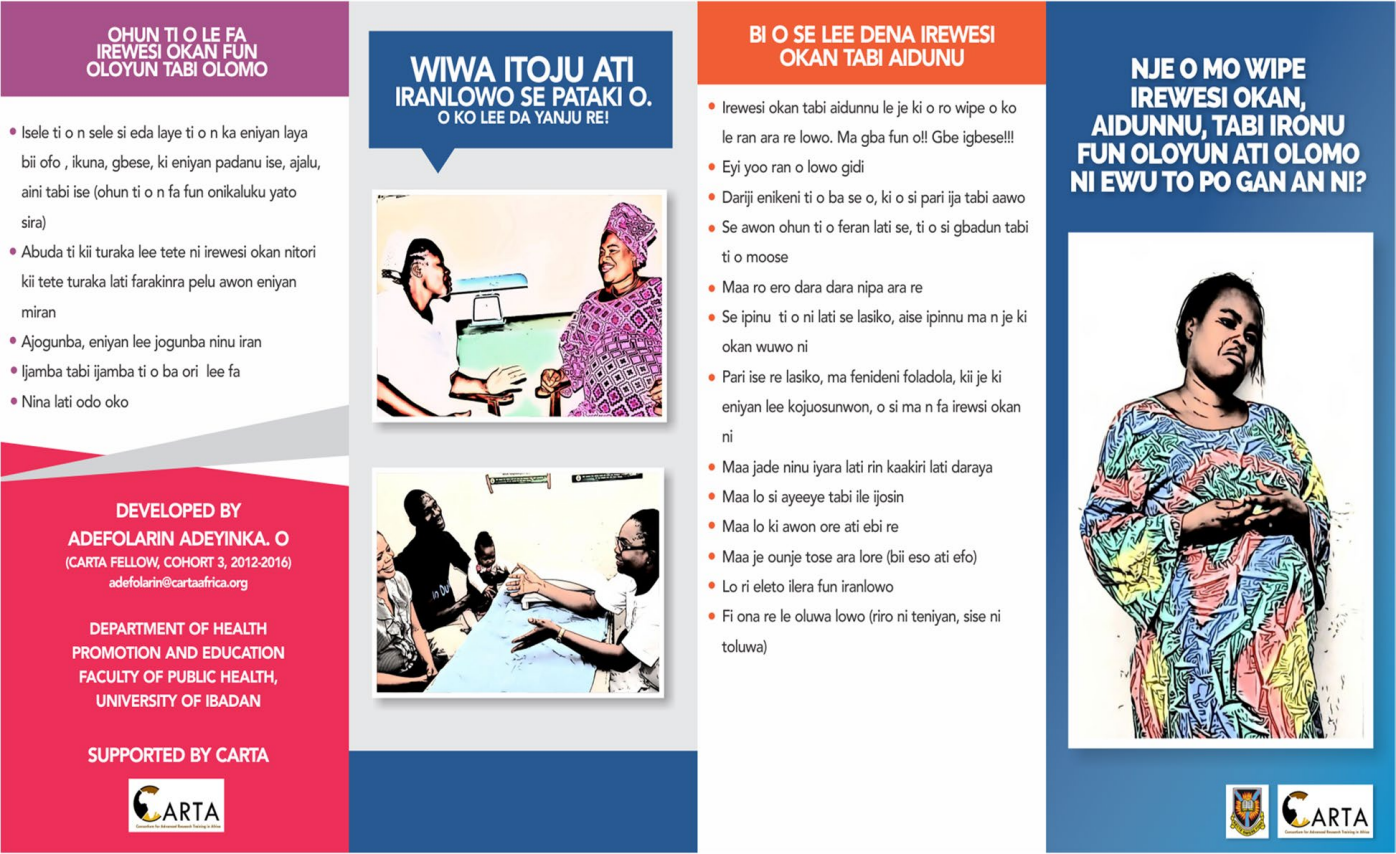
Is the front page of Yoruba leaflet on maternal depression. It shows the definition, symptoms, and consequences of maternal depression

**Fig. 6:**
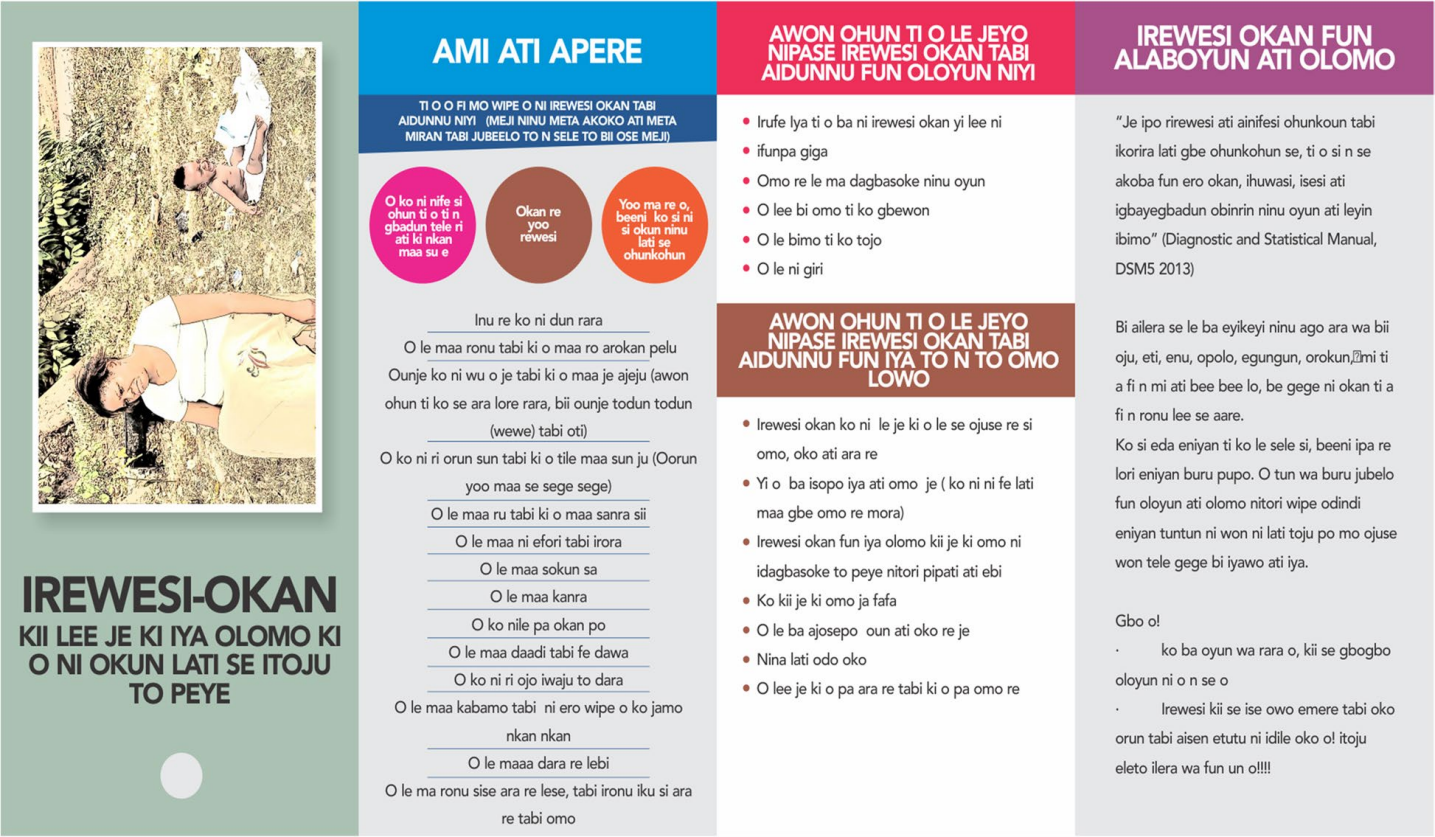
is the back page of Yoruba leaflet on maternal depression. It shows how to prevent and to take control of depression

**Fig. 7:**
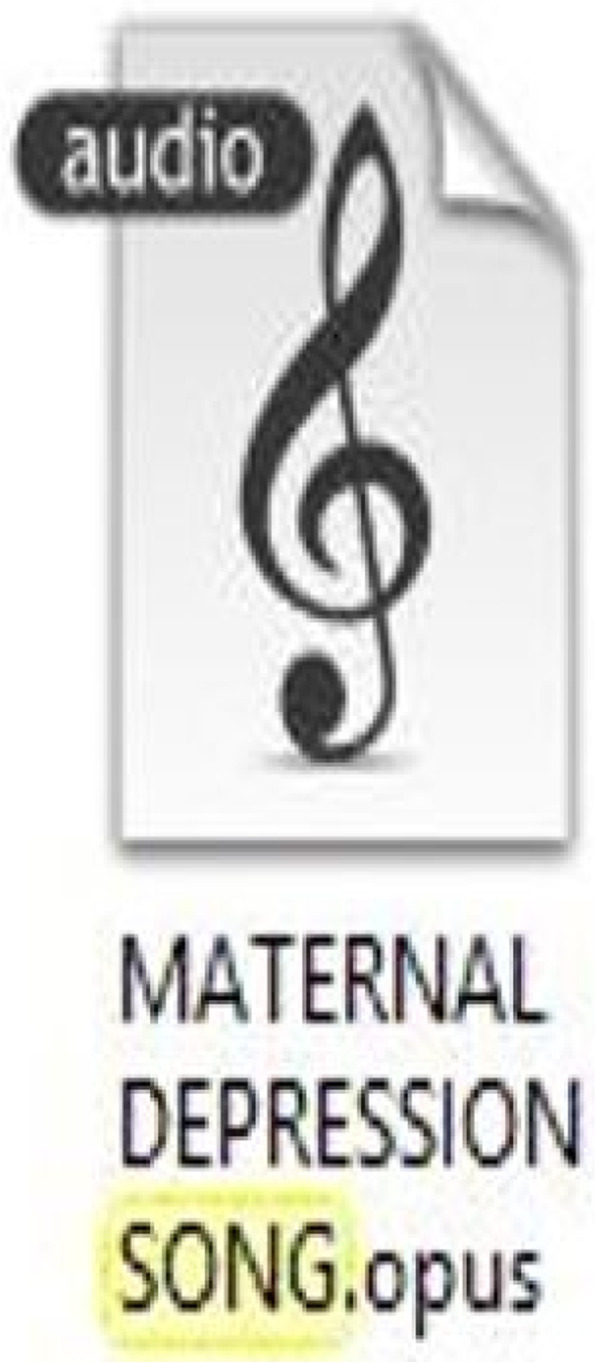
Song on maternal depression. *(This song has no English version; its lyrics were translated from Yoruba language to English language for the purpose of this manuscript)*

Three educational materials (poster, leaflet (Yoruba and English version), and a song (Only Yoruba version) on maternal depression which were developed by researcher A. O are shown in Figs. 1, 2, 3, 4, 5, 6 and 7. Figure 3 shows researcher’s name written at the back page. A written consent for publication has been taken on any identifying images used in this study.

During the process of development of the materials, the maternal-child health clients of fifth to seventh grade pretested them, and evaluated the materials in terms of understanding, clarity, and cultural appropriateness. The content of these materials was built on the concept of health belief model [27] with a clear definition of maternal depression, risk factors which address perceived susceptibility and consequences of maternal depression. The content validation process of these materials, in this present study, was guided by “The best practice for developing and validating scales for health, social and behavior research and [1, 2].

### Content of song 1

Every pregnant woman and nursing mother!!!!

Listen to this instruction, an instruction from the health workers.

Depressed mood in pregnancy and after delivery carries a great risk for mother and child.

Fatigue, isolation, loss of interest in pleasurable things. It carries a great risk for mother and child.

Quickly see the health workers for help.

### Content of song 2

May depression does not disrupt my joy.

I will not allow depression to disrupt my joy.

It is dangerous for me and my child.

I quickly see the health workers for help.

#### Characteristics of study participants and selection procedure

The study participants included health professionals and maternal child health service users/clients (nursing mothers and pregnant women). To be included as a professional participant, the individual should have one of the following professional backgrounds: mental health, maternal-child health, public health nursing in mental health, health promotion, child health and health communication. Professionals who were not bilingual in Yoruba and English language were excluded. Eighteen professionals (six per material) were recruited through snowballing [8]. The recommended number of professional for validation is > 5, the more the better the robustness of rating [1] but it all depends on the availability of professionals in the field of interest. To be included in this study as a maternal-child health users, the client should be accessing routine care at the selected primary health clinics, be able to read Yoruba language and should be available in the waiting area to attend to education sessions. Those who were in a hurry to leave or had a sick child were excluded.

A convenient sample size of 50 was taken that provided a power of 93% at 95% confidence level, margin of error 5%, assuming a loss to follow up of 10% to estimate that 90 ± 20% of women rating the materials as suitable [7].

From two selected LGAs, three clinics with the highest patient load according to the clinic record during the period of the study were purposively selected. The three clinics (A, B, C) were selected from Ibadan North LGA and three clinics (X, Y, Z) from Ibadan Northeast LGA. The clinics names were written and rolled into paper balls that were placed inside a container. Three empty boxes had ‘poster’, ‘leaflet’ and ‘song’ labels written on them. Someone who was not part of the study was invited to randomly pick the first set (A, B, C) into the each of the empty boxes. The same process was repeated for (X, Y, Z). The 50 clients for each material (25 clients from each clinic) were selected randomly as follows: Paper balls labelled “yes” and “no” (50 each) were placed in a box and each eligible client at the clinics was asked to pick one. The clients who picked ‘yes’ each day, and who consented to participate were recruited. This process was repeated on every clinic day until 50 clients for each material in each clinic were recruited.

#### Instrument and data collection procedure

##### Content validation of English and Yoruba maternal depression educational materials by professionals

Content validation of materials by professionals is a requirement in the development of educational materials [2]. Professionals made use of the domains of the Suitability Assessment of Materials (SAM) to rate the English version of each material in Figs. 1,3, and 4 and they utilized the reliable translated Yoruba SAM to rate Yoruba version in Figs. 2,5,6 and 7, 1 month apart. The same six bilingual professionals rated English and Yoruba poster. Another set of six professionals rated English leaflet and Yoruba leaflet. Another set of six professionals rated Yoruba song (song has no English version). The professionals were given copies of the print materials and the tool (Suitability Assessment of Materials) for 1 week. They used the SAM tool to score the English print educational materials on the six domains and submitted. To limit rating bias, after 1 month of submission, the professionals were also given Yoruba translated SAM for 1 week to rate the Yoruba version of the print educational materials, and the Yoruba maternal depression educational was song was given to the designated professionals on their mobile phones.

##### Face validation of Yoruba version maternal depression educational materials by maternal-child health users

Face validity is the “degree that respondents or end users [or lay persons] judge that the items of an assessment instrument are appropriate to the targeted construct and assessment objectives” [1]. Three trained Research Assistants (RAs) were assigned each to the each of the three Yoruba materials in Figs. 2,5,6, and 7, they were trained in consent taking and the administration of SAM tool using cognitive interview. The RAs used the SAM to ask maternal-child health users questions and they recorded the rating/comments on the materials. The process lasted for 1 week on the poster and song, but the leaflet took up to 2 weeks because it has more content and pages.

#### Measurement and data processing

The primary outcome variable for this study is the validity index of the materials (poster, leaflet, and song) as rated by professionals, and the intra-class correlation (agreement) among the professionals’ rating; face validation: Rating of adequacy of all the three educational materials with focus of analysis on the association bewtween content, literacy demand, cultural appropriateness, and sociodemographic characteristics among the maternal child health service users; and the agreement (intra-class correlation) between professionals’ ratings and clients’ rating.

##### Validity index measurements

This validity index was computed on the professionals’ rating of the educational materials with use of the 6 items on SAM (content, literacy, graphics, layout, simulation, and cultural appropriateness) as applicable to each material. The SAM originally has a rating of 2 for superior, 1 for adequate and 0 for not suitable. For the computation of validation 1, and 2 are regarded as 1 = adequate, and 0 = not adequate [8]. Validity index was calculated by formulas: I-CVI = Number of professionals who rated an item as adequate/the total number of professionals; S-CVI/UA = I-CVI rated 1/total no of items [28]. There are two kinds of Content Validity Index (CVI) [29]: Item level- Content Validity Index (I-CVI) and Scale level – Content Validity Index Average (S-CVI/Ave) or Scale level- Content Validity Index/ Universal Agreement (S-CVI/UA). The I-CVI = the number of professionals who rated the item 1(not suitable) or 2 (suitable) divided by the total number of professionals. S-CVI/UA or S-CVI/Ave are both scale level CVI with different formulas. S-CVI/UA is calculated by adding all items with I-CVI equal to 1 divided by the total number of items. On the other hand, S-CVI/Ave is calculated by taking the sum of the I-CVIs divided by the total number of items. A domain of the education material with excellent content validity should be composed of I-CVIs ≥0.78, S-CVI/UA ≥0.8 or S-CVI/Ave ≥0.9.

##### Face validation among maternal-child health clients

Descriptive analysis was used to analyze the frequencies of the socio demographic characteristics of the client participants and their rating on the suitability assessment of the Yoruba version of the materials. The SAM score rates ≥70% (Superior: 2); ≥40–70% (adequate: 1) and ≤ 40% (not suitable:0). Fisher exact test was used to assess the association of the sociodemographic characteristics of clients and suitability rating on content, literacy demand, and cultural appropriateness with *p*-value of significance set at 0.05. According to [30], these three domains: content, literacy demand, and cultural appropriateness of SAM are regarded as the most important domains. If all other domains are scored superior, but these three domains are not suitable, the materials are expected to be revised.

##### Inter-rater agreement among professionals

Inter-rater reliability analysis was carried out using the intra-class correlation (ICC) within the ratings of the six professionals for each material (English and Yoruba version) using SPSS. The agreement among professionals on each material is an indication of validity [25]. The ICC > 0.8 shows very strong agreement.

##### Inter-rater agreement among the professionals and clients’ ratings

Inter-rater reliability using intra- class correlation (ICC) was carried out on the rating of each Yoruba material among the professionals and clients using SPSS 25. Six clients for each material were randomly selected from the clients’ data. This is possible because both groups utilized SAM tool to rate the materials. The ICC > 0.8 shows very strong agreement. In the study of [31], they carried out inter-rater reliability using intra-class coefficient to check the agreement between the parent-teacher pairs in their ratings of pupils. This present study also utilized ‵t‵ test (*p* < 0.05 signifies no statistical difference), and Pearson correlation (*r* < 0.5 is a weak correlation while *p* > 0.05 signifies no linear relationship exist) to analyze the agreement between professionals and clients’ ratings.

#### Ethical consideration

This study is a part of parent study “Effect of training and supervision of maternal depression inclusive health education delivery among primary health care workers in Ibadan, Nigeria” which received an ethical review approval form the Ministry of Health, Oyo state Nigeria ref. no AD 13/479/2016. Written consents were taken from the professionals and the maternal child health service users who participated in the study. The consent contained the information about the study and voluntary nature of participation. The consent also assured participants of confidentiality and data protection. No names of individuals were collected, but codes were used as identifiers on the measuring instruments.

## Results

### Sociodemographic characteristics of professionals for content validation of maternal depression education materials

Table 1 shows the sociodemographic characteristics of 18 professionals who participated in the validation of educational materials on maternal depression. Half were aged > 40 years. Their mean age is 44.3 ± 6.0 years for poster, 39.8 ± 7.2 years for leaflet and 43.8 ± 8.4 years. 94.4% are of the Yoruba tribe, 88.9% were health workers, while 11.1% were in academia. Their professional background included mental health 3(16.6%), Child health 3(16.6%), Health promotion 3(16.6%), Public health 3(16.6%), maternal child health 3(16.6%) and health communication 3(16.6%) for each material.

**Table 1:** The sociodemographic information of professionals who participated in the validation of educational materials

	English &Yoruba version		English &Yoruba version		Yoruba	
	Poster		Leaflet		Song	
**Mean of all professionals**		41.6 ± 7.2				
**Mean age**	44.3 ± 6.0 years		39.8 ± 7.2 years		43.8 ± 8.4 years	
**Age in years**						
< 40	03	50.0	04	60.0	02	33.3
> 40	03	50.0	02	40.0	04	66.7
Total	6	100.0	6	100.0	6	100.0
**Educational Qualification**						
B.ED^a^	1	16.7				
FWACS^b^	1	16.7				
MBBS^c^	1	16.7			2	33.3
MPH^d^	1	16.7	2	33.3	1	16.7
MSC^e^	1	16.7	2	33.3	2	33.3
RN^f^	1	16.7	2	33.3	1	16.7
Total	6	≈100	6	≈100	6	≈100
**Professional**						
Mental health	1	16.7	1	16.7	1	16.7
Child health	1	16.7	1	16.7	1	16.7
mental health	1	16.7	1	16.7	1	16.7
Public health	1	16.7	1	16.7	1	16.7
Health promotion	1	16.7	1	16.7	1	16.7
Maternal child health matron	1	16.7	1	16.7	1	16.7
Total	6	≈100	6	≈100	6	≈100
**Marital Status**						
married	5	83.3	6	100.0	5	83.3
widowed	1	16.7				
single	6	100.0			1	16.7
Total	6		6		6	100.0
**Work experience**						
3-5 yrs	2	33.3				
6-9 yrs	0				2	33.3
> 10 yrs	4	66.7	6	100.0	4	66.7
Total	6	100.0	6	100.0	6	100.0
**Work type**						
Health workers	5	83.3	4	66.7	4	66.7
Lecturer	1	16.7	2	33.3	2	33.3
Total	6	100.0	6	100.0	6	100.0
**Tribe**						
Yoruba	5	83.3	6	100.0	6	100.0
Ibo	1	16.7				
Total	6	100.0	6	100.0	6	100.0
**Religion**						
Christianity	6	100.0	6	100.0	6	100.0
Total	6		6		6	

BED^a^Bachelor of Education, FWAPC^b^-Fellow West Africa College of Physician (FWACP) West Africa Psychiatry College, MBBS^c^: Bachelor of Medicine and Surgery, MPH^d^- Master of Public Health, MSc^e^-Master of Science, RN^f^-Registered Nurse

### Socio demographic characteristics of maternal child health clients who evaluated educational materials

Table 2 show the sociodemographic characteristics of maternal child health clients who participated in the rating of the suitability assessment of poster, song, and leaflet on maternal depression. Their mean age is 30.7 ± 5.4 years for poster, 31.3 ± 5.2 years for leaflet and 29.0 ± 5.1 years for song. Fifty participants rated each material. The category of pregnant women and nursing mothers were distributed equally across the 3 materials. Poster had all Yoruba tribe participants while song and leaflet had other tribes represented but majority are Yorubas 88 and 86% respectively. Majority of the participants achieved post-secondary school educational level (post grade 9) respectively as follows for Poster 38(76%), leaflet 30 (60%) and song 28(56%).

**Table 2:** Sociodemographic characteristics of maternal-child health clients who evaluated maternal depression educational materials

Socio demographic characteristics of respondents	Participants for poster assessment N (50) %		Participants for leaflet assessment N (50) %		Participants for song assessment N (50) %	
**Mean age in years**	30.7 ± 5.4		31.3 ± 5.2		29.0 ± 5.1	
**Age range in years**						
20–30	31	62.0	25	50.0	32	64
31–49	19	38.0	25	50.0	18	36
Total	50	100.0	50	100.0	50	100
**Category of women**						
Pregnant	25	50.0	25	50.0	25	50.0
Nursing mother	25	50.0	25	50.0	25	50.0
Total	50	100.0	50	100	50	100
**Religion**						
Christianity	24	48.0	24	48.0	26	52.0
Islam	26	52.0	26	52.0	24	48.0
Total	50	100.0	50	100	50	100
**Highest level of education**						
< Secondary school	12	24.0	20	40.0	22	44.0
> Secondary school	38	76.0	30	60.0	28	56.0
Total	50	100	50	100	50	100
**Occupation**						
Non-Civil servant	30	62.0	34	68.0	31	62
Civil servant	19	38.0	16	32.0	19	38
Total	50	100	50	100	50	100
**Ethnic group**						
Yoruba	50	100.0	43	86.0	44	88.0
Non-Yoruba	0	0	07	14.0	06	12.0
Total	50	100	50	100	50	100

### The rating of adequacy of the content, literacy demand and cultural appropriateness of the Yoruba version educational materials among the maternal-child health clients

Table 3 shows fisher exact analysis of the socio demographic background of the mater-child health client participants, and the domains of content, literacy demand and cultural appropriateness. The clients rated all the materials as superior: Poster 44 (88%), leaflet 45(90%), and song 50(100%). There is no significant relationship between the domains of content, literacy demand, cultural appropriateness and sociodemographic characteristics of the participants across poster, leaflet, and song materials. Regardless of the status of the socio demographic characteristics of the clients, they rated all the materials as superior. On the comment column of the SAM, clients made no suggestion on the improvement of the materials, but they expressed what they liked about the materials as follows. “*The leaflet needs patience to read it. It has a sentence which addresses mothers and mother in-laws, that they should be supportive of any depressed mother by taking them to the hospital. The leaflet has a good picture showing husband and wife going to see a nurse. As a take home material, the picture will teach men to follow their wives to the hospital”.* For all the materials they said*. “They enlighten mothers to know that depression is a sickness not something one can get over with time. The help seeking part of all the materials make us to know that hospital can treat depression very well”.*

**Table 3:** Association of sociodemographic factors with the ratings of the content, literary, and cultural appropriateness domains

POSTER	Content ***N*** = 50	Fisher exact	Literacy demand ***N*** = 50	Fisher exact	Cultural appropriateness ***N*** = 50	Fisher exact
	Superior rating		Superior rating		Superior rating	
**Age**		1.00		1.00		0.4
< 30 years	26		24		30	
> 30 years	18		17		19	
**Education**		0.17		0.58		1.0
< Secondary school	14		13		14	
> Secondary school	30		28		35	
**Occupation**		1.00		1.00		0.34
Non-Civil servant	29		27		33	
Civil servant	15		14		16	
**Category of women**		0.67		0.46		–
Pregnant woman	22		22		25	1.0
Nursing mother	22		19		24	
**LEAFLET**						
**Age**		0.35			25	–
< 30 years	24		25		25	
> 30 years	21		23			
**Education**		1.00		1.00		–
< Secondary school	17		18		19	
> Secondary school	28		30		31	
**Occupation**		0.27		1.00		–
Non-Civil servant	43		45		47	
Civil servant	2		3		3	
**Category of women**		0.64		1.00		–
Pregnant woman	27		28		29	
Nursing mother	18		20		21	
**SONG**						
**Age**		–		0.64		–
< 30 years	30		27		30	
> 30 years	20		19		20	
**Education**		–		–		–
< Secondary school	22		20		22	
> Secondary school	44		29		28	
**Occupation**		–		–		–
Non-Civil servant	28		25		28	
Civil servant	22		21		22	
**Category of women**		–		0.61		–
Pregnant woman	25		24		25	
Nursing mother	25		22		25	

Level of significance is set at 0.05

### Validity index of English and Yoruba version of poster, leaflet, and the Yoruba version of song among professionals

Table 4 shows the Item Level Content Validity Index (I-CVI) for poster, leaflet, and song by different groups of six bilingual professionals who rated each material. The ratings of the professionals for the English and Yoruba version of leaflet and poster are the same despite that the rating was carried out at 1 month interval. Song has the highest I-CVI of 1(excellent content validity). The Scale level content validity/ Universal Agreement S-CV/AU among the professionals is 0.83 (> 0.8) for all the materials (excellent content validity). The professionals rated the suitability of all the materials as superior, and on the scale of 0–10 (as stated on the SAM tool), they got an average rating of 8 for poster, 9 for song and 8 for leaflet.

**Table 4:** Item content validity index of English and Yoruba poster, leaflet and Yoruba song materials from professionals’ rating

Materials/SAM Domains/Professionals	1	2	3	4	5	6	A	B	I-CVI = A/B	1	2	3	4	5	6	A	B	I-CV = A/B
	ENGLISH VERSION								YORUBA VERSION									
**LEAFLET**																		
Content	x	x	x	x	x	x	6	6	1	x	x	x	x	x	x	6	6	1
Literacy demand	x	x	x	x	x	x	6	6	1	x	x	x	x	x	x	6	6	1
Graphics	x	x	x	x	x	x	6	6	1	x	x	x	x	x	x	6	6	1
Layout and topography	x		x	x	x	x	5	6	0.83	x		x	x	x	x	5	6	0.83
Learning simulation	x	x	x	x	x	x	6	6	1	x	x	x	x	x	x	6	6	1
Cultural appropriateness	x	x	x	x	x	x	6	6	1	x	x	x	x	x	x	6	6	1
Total I-CVI= total A/ total B							34	36	0.94							34	36	0.94
***S-CVI/UA***									0.83									0.83
**POSTER**																		
Content	x	x	x	x	x	x	6	6	1	x	x	x	x	x	x	6	6	1
Literacy demand	x	x	x	x	x	x	6	6	1	x	x	x	x	x	x	6	6	1
Graphics	x	x	x	x	x	x	6	6	1	x	x	x	x	x	x	6	6	1
Layout and topography	x	x	x	x		x	5	6	0.83	x	x	x	x		x	5	6	0.83
Learning simulation	x	x	x	x	x	x	6	6	1	x	x	x	x	x	x	6	6	1
Cultural appropriateness	x	x	x	x	x	x	6	6	1	x	x	x	x	x	x	6	6	1
Total I-CVI= total A/ total B							34	36	0.94							34	36	0.94
***S-CVI/UA***									0.83									0.83
**SONG**																		
Content										X	x	x	x	x	x	6	6	1
Literacy demand										X	x	x	x	x	x	6	6	1
Learning simulation										X	x	x	x	x	x	6	6	1
Cultural appropriateness										X	x	x	x	x	x	6	6	1
Total I-CVI= total A/ total B																24	24	1
***S-CVI/UA***																		1

S-CVI/UA = Item-CVI rated as 1/total no of items. A is the number of professionals who rated material as adequate. B is the total number of professionals

### Interrater agreement among mental health professionals on the English and Yoruba versions of print educational materials and Yoruba version of song using intra-class correlation (ICC)

The mental health professionals rated the English and Yoruba version of poster and leaflet the same, hence interrater agreement for the English version is the same for the Yoruba version of the print materials. Table 5 shows the agreement among the raters for the three materials is statistically significant *p* < 0.01. All the raters had inter-rater agreement of ICC above 0.75 on the adequacy of the leaflet, while there was a weak inter-rater agreement for poster. There was no absolute agreement among all the raters on the adequacy of the poster. The agreement between expert 2 and 5; 6 and 1,2,3,4, have an intra-class coefficient < 0.6. The song and leaflet material have no disagreement in the suitability rating of all the professionals. The agreement across all the professionals on leaflet and song is very strong with ICC ≥8.0.

**Table 5:** Inter-rater agreement among the professionals on the rating of English and Yoruba poster and leaflet, and Yoruba version of song using intra-class coefficient

	Professional 1	Professional 2	Professional 3	Professional 4	Professional 5	Professional 6
**Intra-class coefficient matrix for English and Yoruba leaflet**						
Professional 1	1.000	.866	.911	.987	.903	.974
Professional 2	.866	1.000	.817	.896	.795	.897
Professional 3	.911	.817	1.000	.939	.984	.972
Professional 4	.987	.896	.939	1.000	.909	.982
Professional 5	.903	.795	.984	.909	1.000	.965
Professional 6	.974	.897	.972	.982	.965	1.000
**Intra-class coefficient matrix for English and Yoruba poster**						
Professional 1	1.000	.908	.856	.906	.823	.133
Professional 2	.908	1.000	.610	.732	.572	.266
Professional 3	.856	.610	1.000	.746	.765	.143
Professional 4	.906	.732	.746	1.000	.965	−.125
Professional 5	.823	.572	.765	.965	1.000	−.088
Professional 6	.133	.266	.143	−.125	−.088	1.000
**Intra-class coefficient matrix for Yoruba song**						
	Professional 1	Professional 2	Professional 3	Professional 4	Professional 5	Professional 6
Professional 1	1.000	.974	.843	1.000	.953	.974
Professional 2	.974	1.000	.793	.974	.887	.961
Professional 3	.843	.793	1.000	.843	.704	.700
Professional 4	1.000	.974	.843	1.000	.953	.974
Professional 5	.953	.887	.704	.953	1.000	.971
Professional 6	.974	.961	.700	.974	.971	1.000

### Inter-rater agreements between professionals and clients on Yoruba poster, leaflet, and song

Table 6 shows the agreement of rating of randomly selected six clients for each of Yoruba poster, Leaflet and Song with the rating of the six fixed professionals for each of the Yoruba poster, leaflet, and song. It shows no inter-rater agreement between the ratings of the professionals and clients on the poster with an intraclass-correlation of 0.30, and the *p* value is >0.05. On the leaflet and song, there is no agreement in their ratings because the intra-class correlation coefficient is negative, and the *p* value >0.05 is not significant.

**Table 6:** Inter-rater agreement between the rating of professionals and the ratings of clients on Yoruba poster, leaflet, and song

PARTICIPANTS = 6	POSTER RATING		ICC	***P*** value	LEAFLET RATING		ICC	***P*** value	SONG RATING		ICC	***P*** value
	Lower bound	Upper bound			Lower bound	Upper bound			Lower bound	Upper bound		
**6 Professionals ‘rating**	−0.126	0.821	**0.301**	**0.064**	−0.122	− 0.054	**− 0.094**	**0.958**	*1 (10)*	*1 (90)*	**−0.408**	**0.619**
***6Clients’rating***	−0.126	*0.821*			*−0.122*	*−0.054*			*1 (10)*	*1 (90)*		

### Independence t test on professionals’ rating and clients’ ratings of Yoruba version of educational materials

Table 7 shows independent ‵t′ test shows that is a statistical difference between the ratings of professionals and clients on poster and leaflet, while there is no significant difference found in their ratings on song. Meaning professionals and clients rated song the same way.

**Table 7:** Independence t test on professionals’ rating and clients’ ratings on Yoruba poster, leaflet, and song

Materials	Raters	Mean Scores	SD	Mean difference	t	***P*** value
**Poster**	**Professionals**	27.2	3.81	−9.333	−3.96	0.003
	**Clients**	36.5	4.32			
**Leaflet**	**Professionals**	27.2	3.06	−14.833	−9.39	0.000
	**Clients**	42.0	2.36			
**Song**	**Professionals**	26.3	1.75	0.833	0.405	0.694
	**Clients**	25.5	4.72			

Table 8 shows the Pearson correlation between the ratings of the professional and the ratings of the clients. On the poster, there is no significant relationship in their ratings *p* = 0.174. On the leaflet, there is a significant relationship in the ratings of the professionals and the clients *p* < 0.05, but the correlation is a high negative linear relationship − 0.837. On the song, there is no significant relationship in the ratings of the two groups *p* = 0.679.

**Table 8:** Pearson correlation of six professionals and randomly selected six clients’ ratings of Yoruba version of educational materials

EDUCATIONAL MATERIALS	YORUBA POSTER		Pearson Correlation	***P*** value
RATERS	PROFESSIONALS	CLIENTS	0.636	0.174
	*N* = 6	*N* = 6		
1	26.00	30.00		
2	21.00	32.00		
3	26.00	40.00		
4	28.00	39.00		
5	30.00	39.00		
6	32.00	39.00		
	**YORUBA LEAFLET**			
	PROFESSIONALS	CLIENTS	−0.837	0.035
	*N* = 6	*N* = 6		
1	32.00	39.00		
2	25.00	43.00		
3	23.00	44.00		
4	28.00	39.00		
5	28.00	42.00		
6	27.00	43.00		
	**YORUBA SONG**			
	**PROFESSIONALS**	**CLIENTS**		
1	27.00	16.00	−0.218	0.679
2	23.00	28.00		
3	27.00	26.00		
4	26.00	27.00		
5	28.00	28.00		
6	27.00	28.00		

## Discussion

This study validated English version of already developed poster and leaflet, and Yoruba version of the poster, leaflet, and song on maternal depression. It also shows the content validation process. All these materials have excellent validity index > 0.8 based on professionals’ ratings. Likewise, based on clients’ evaluation, > 80% rated each material’s suitability as superior using the same SAM tool as the professionals. Sociodemographic characteristics did make clients’ ratings differ on the SAM’s domains of content, literacy demand and cultural appropriateness with the Fishers exact analysis. These are the domains which the author of SAM regards as the most important in clients’ evaluation.

We did not come across any other study in Nigeria that validated such education materials as ours. Meanwhile, a systematic review on the effect of print educational materials on professional practice and health outcome found only one study that validated print educational material in low- and middle-income countries [10]. In the developed countries, many studies are available, of which one of them was used as a guide for our study. The study is found in Brazil, and it combined the development and validation process of educational material on nutrition in pregnancy in one study [7]. Although, our study is only based on validation process not the development part of the educational materials. The authors of nutrition in pregnancy booklet utilized SAM’s rating and found validity index > 0.8. Other studies that utilized SAM and reported findings which are consistent with ours include a study in Portugal on adolescents [8] and in Washington [32], they both achieved excellent validity index (> 0.8) also.

The process of content validation in this study is consistent with other studies [7, 8], but different from two other study which made use of four-point Likert scale as against SAM tool [9] and the one which made use of Delphi techniques among expert or judges [25]. These two studies utilized binomial test and Kappa to determine the agreement in the ratings of the experts. In all these studies, experts or judges or professionals were engaged in the validation process because they understand the phenomenon of focus [2]. In our own study, we made use of professionals who are stakeholders of maternal depression education; child health, health promotion, mental health, public health in mental health, maternal health-child health, and health communication professionals. The agreement among these experts is the main thrust of content validation [2]. Our study measured inter-rater agreement on the Scale-Item Level Content Validity Index/Universal Agreement (S-ICVI/AU). The outcome is > 0.8 for each material, which signifies excellent agreement. This process is consistent with [7] in Brazil on nutrition in pregnancy booklet. This Brazilian study likewise made use of S-ICVI/AU and found excellent agreement> 0.8. We also utilized inter-rater reliability of the professionals’ ratings using intra-class coefficient (ICC) which shows that their agreement is weak on the adequacy of the poster< 0.6. The ICC for leaflet and song shows excellent coefficient, the agreement of ratings among the professional is strong; ICC > 0.8. This same inter- rater reliability was used [33] in Brazil on prevention of metabolic syndrome among adolescent education material. The study considered inter-rater agreement among the professionals like ours and excellent agreement > 0.8 was achieved. It is not enough to utilize S-ICVI/AU alone, the inter-rater reliability shows the clear-cut consistencies of the ratings on who agrees with who among the professionals.

The evaluation of materials by the end users is as important as that of the professionals. If the target users find materials not adequate during face validation, the process of expert validation could be a waste [9, 25], because the materials are made for the consumption of the users. In our study, the professionals and the clients utilized the same instrument to rate the educational materials, but their scores show that clients rated materials higher than professionals. The proportion of ratings among the users was considered as face validation. However, the possibility of an agreement in the ratings of the professionals and the clients was examined. Six clients out of the fifty clients were randomly selected for each material to compare with the six professionals for each material. This process of examining the agreement between professionals and clients' ratings is a rare occurrence in the field of educational material development, but our study attempted it. The comparison of the professionals’ and clients' ratings shows a statistical difference on poster and leaflet, but on the song the difference is not statistically significant on the ′t‵ test analysis. On the interrater reliability, the intra class coefficient does not show any agreement across all the materials among the two groups of raters. Pearson correlation analysis only shows a negative linear relationship on the ratings of the leaflet. It shows no relationship on the ratings of the poster and song. This may imply that the translated SAM tool has a potential for difference when two groups of raters with different socio-demographic background make use of it for rating educational materials.

Similarly, lack of agreement was spotted in the findings of [25] between target users and the professionals’ opinions. They had different opinions regarding educational materials for orthognathic surgery. Hence, the authors complied with the users’ opinion. Although, the author noted that over simplicity may have occurred in the ratings of clients. This over simplicity could explain the much higher rating of clients than the professionals’ in our own study also, but our study reckons with the judgement of professionals (restrictive rating of the materials) because of the sensitivity of the condition, 'maternal depression'. However, a study utilized ELAN, a German tool for rating children’s vocabulary. The teacher group and the parent group with similar educational background rated children vocabulary, and agreement was found in their rating on the interrater reliability [31]. High Pearson correlation was also found on their ratings. This ELAN study throws light on the possibility of socio-demographic characteristics (educational background) playing a major role in agreement or disagreement in the ratings among two groups.

The highest qualification of the professionals in our study was FWACP (Fellow of West Africa College of Physician) and the lowest is Registered Nurse. All the professionals achieved tertiary education, and they have the technical know-how in their fields of practice. There is less disparity in the reliability of their ratings across the three materials. These findings agree with those studies which utilized SAM ratings among nurses with minimum of 15 years experience in women’s health or related field as professional raters [7], and other study with participants having higher qualifications such as professors and PhD holders as professional raters [8]. Their tertiary educational background and expertise in the field of interest contributed to the similarities in their ratings. The minimum educational qualification of the client raters in our study is 9th grade. Hence, this could be the reason for the disparity. However, the clients found the materials acceptable.

### Limitation and strength of this study

Our study has some limitations. All print educational materials are expected to reach the readability level of fifth grade educational qualification [34–36] . Majority (76%) of our clients had post-secondary education (post ninth grade), more women tend to achieve schooling beyond 5th grade in the study area. However, the Yoruba translated versions of the materials have taken care of the low literate people, and the development process of the materials which is not reported in this study involved maternal-child health clients with fifth to seventh grade. This study has the strength of showcasing process of validation of educational materials available in local language. It also provides evidence to the needless effort of comparing the ratings of people with different education background (clients and professionals) because their ratings will most likely not agree.

## Conclusion

Our study found that the poster, leaflet, and song educational materials on maternal depression have excellent content validity among professionals and acceptable by clients. These materials can therefore be recommended for use in maternal child health clinics for educating clients. Researchers intending to develop educational materials in Africa can leverage this validation process for developing locally relevant materials.

## Data Availability

The datasets used and/or analyzed during the current study available from the corresponding author on reasonable request.

## Abbreviations

SAM: Suitability Assessment Materials
I-CVI: Item level- Content Validity Index or Scale level- Content Validity Index
S-CVI/Ave: Scale level – Content Validity Index/ Average
S-CVI/UA: Scale level – Content Validity Index/Universal Agreement

## References

[1] Haynes SN, Richard DCS (1995). Content validity in psychological assessment: a functional approach to concepts and methods introduction to content validity.

[2] Boateng GO, Neilands TB, Frongillo EA, Melgar-Quiñonez HR, Young SL (2018). Best practices for developing and validating scales for health, social, and behavioral research: a primer. Front Public Health.

[3] Lynn MR (1986). Determination and quantification of content validity. Nurs Res.

[4] Cohen JA, Fischer JS, Bolibrush DM, Jak AJ, Kniker JE, Mertz LA (2000). Intrarater and interrater reliability of the MS functional composite outcome measure. Neurology.

[5] Wynd CA, Schmidt B, Schaefer MA (2016). Two quantitative approaches for estimating content validity. West J Nurs Res.

[6] Linstone HA, Turoff M (2011). Delphi: a brief look backward and forward. Technol Forecast Soc Chang.

[7] de Oliveira SC, de Oliveira Lopes MV, Fernandes AFC (2014). Development and validation of an educational booklet for healthy eating during pregnancy. Revista Latino-Americana de Enfermagem.

[8] Holanda De Moura I, Rodrigues Da Silva AF, Do A, Santo E, Rocha H, Helena L (2017). Construction and validation of educational materials for the prevention of metabolic syndrome in adolescents 1. Rev Latino-Am Enfermagem.

[9] Hurst CS, Hagensee ME, Ahmed SA, Smith JS (2015). Validation of educational tools for use in a human papillomavirus intervention study. Cancer Oncol Res.

[10] Summary S (2016). What are the effects of printed educational materials on professional practice and healthcare outcomes?.

[11] Rowe AK, Rowe SY, Peters DH, Holloway KA, Chalker J, Ross-Degnan D (2018). Effectiveness of strategies to improve health-care provider practices in low-income and middle-income countries: a systematic review. Lancet Glob Health.

[12] Ciapponi A, Lewin S, Herrera CA, Opiyo N, Pantoja T, Paulsen E, Rada G, Wiysonge CS, Bastías G, Dudley L, Flottorp S, Gagnon MP, Garcia Marti S, Glenton C, Okwundu CI, Peñaloza B, Suleman F, Oxman AD. Delivery arrangements for health systems in low-income countries: an overview of systematic reviews. Cochrane Database Syst Rev. 2017;(9):CD011083. 10.1002/14651858.CD011083.pub2 PMC5621087 28901005

[13] Ogundeji MO (Martins O, Adeniyi JD, Osungbade KO, Arulogun OS. Primary health care in Nigeria: history and development. Ibadan University Press. ISBN 978-978-8456-71-1.

[14] Adefolarin AO, Arulogun OS (2018). Need assessment for health education service provision on maternal depression among primary health care service providers. Arch Basic Appl Med.

[15] Dadi AF, Miller ER, Bisetegn TA, Mwanri L. Global burden of antenatal depression and its association with adverse birth outcomes: an umbrella review. BMC Public Health. 2020;20(1):173. 10.1186/s12889-020-8293-9. 10.1186/s12889-020-8293-9 PMC7001252 32019560

[16] Stuart-Parrigon K, Stuart S (2014). Perinatal depression: an update and overview. Curr Psychiatry Rep.

[17] Shidhaye P (2014). Maternal depression: a hidden burden in developing countries. Ann Med Health Sci Res.

[18] Adefolarin AO, Arulogun OS (2018). Perception of mothers and selected informal maternity caregivers regarding maternal depression in two communities of Ibadan in Nigeria. Arch Basic Appl Med.

[19] Eames S, McKenna K, Worrall L, Read S (2003). The suitability of written education materials for stroke survivors and their carers. Top Stroke Rehabil.

[20] Hoffmann T, Ladner Y (2012). Assessing the suitability of written stroke materials: an evaluation of the interrater reliability of the suitability assessment of materials (SAM) checklist. Top Stroke Rehabil.

[21] Shieh C, Hosei B (2008). Printed health information materials: evaluation of readability and suitability. J Community Health Nurs.

[22] Shoemaker SJ, Wolf MS, Brach C (2014). Development of the patient education materials assessment tool (PEMAT): a new measure of understandability and actionability for print and audiovisual patient information. Patient Educ Couns.

[23] Ryan L, Logsdon MC, Mcgill S, Stikes R, Senior B, Helinger B (2014). Evaluation of printed health education materials for use by low-education families. J Nurs Scholarsh.

[24] Wallengren C, Rosengren K, Sawatzky R, Ohlen J (2018). Challenges when translating and culturally adapting a measurement instrument: the suitability and comprehensibility of materials (SAM+CAM). Glob Qual Nurs Res.

[25] Sousa CS, Turrini RNT (2012). Creating and validating educational material for patients undergoing orthognathic surgery. Asian Nurs Res.

[26] Ajayi IO, Falade CO, Bamgboye EA, Oduola AMJ, Kale OO. Assessment of a treatment guideline to improve home management of malaria in children in rural South-West Nigeria. Malar J. 2008:7–11. 10.1186/1475-2875-7-24 PMC2268701 18226272

[27] Rosenstock IM, Strecher VJ, Becker MH. Social learning theory and the health belief model. Health education quarterly. 1988;15(2):175–83. 10.1177/109019818801500203 3378902

[28] Zamanzadeh V, Ghahramanian A, Rassouli M, Abbaszadeh A, Alavi-Majd H, Nikanfar A-R (2015). Design and implementation content validity study: development of an instrument for measuring patient-centered communication. J Caring Sci.

[29] Shi J, Mo X, Sun Z (2012). Content validity index in scale development. J Cent South Univ (Med Sci).

[30] Larsson H, Tegern M, Monnier A, Skoglund J, Helander C, Persson E, Malm C, Broman L, Aasa U. Content Validity Index and Intra- and Inter-Rater Reliability of a New Muscle Strength/Endurance Test Battery for Swedish Soldiers. PLoS One. 2015;10(7):e0132185. 10.1371/journal.pone.0132185. 10.1371/journal.pone.0132185 PMC4503674 26177030

[31] Stolarova M, Wolf C, Rinker T, Brielmann A (2014). How to assess and compare inter-rater reliability, agreement and correlation of ratings: an exemplary analysis of mother-father and parent-teacher expressive vocabulary rating pairs. Front Psychol.

[32] Kaphingst KA, Kreuter MW, Casey C, Leme L, Thompson T, Cheng MR, Jacobsen H, Sterling R, Oguntimein J, Filler C, Culbert A, Rooney M, Lapka C. Health Literacy INDEX: development, reliability, and validity of a new tool for evaluating the health literacy demands of health information materials. J Health Commun. 2012;17 Suppl 3:203–21. 10.1080/10810730.2012.712612. 10.1080/10810730.2012.712612 23030571

[33] Construction and validation of educational materials for the prevention of metabolic syndrome in adolescents. Available from: http://www.scielo.br/scielo.php?script=sci_arttext&pid=S0104-11692017000100383. Cited 28 Nov 2019. 10.1590/1518-8345.2024.2934 PMC5635697 29020125

[34] Doak CC, Doak LG, Root JH (1996). SAM: suitability assessment of materials for evaluation of health-related information for adults. Teaching Patients with Low Literacy Skills.

[35] UNESCO (2010). World Data on Education Données mondiales de l ’ éducation Datos Mundiales de Educación.

[36] Doak CC, Doak LG, Root JH (2007). Teaching patients with low literacy skills | health literacy studies | Harvard T.H. Chan School of Public Health.

